# Supplementary material for: Lectin and E. coli Binding to Carbohydrate-Functionalized Oligo(ethylene glycol)-Based Microgels: Effect of Elastic Modulus, Crosslinker and Carbohydrate Density
Source: Molecules. 2021 Jan 7;26(2):263. doi: 10.3390/molecules26020263 (PMC7825725; doi:10.3390/molecules26020263)
Supplement: Supplementary file 1 [file molecules-26-00263-s001.pdf]

Supporting Information

Lectin and *E. coli* binding to carbohydrate functionalized oligo(ethylene glycol)-based microgels: Effect of elastic modulus, crosslinker and carbohydrate density

Fabian Schröer [1], Tanja J. Paul [1], Dimitri Wilms [1], Torben H. Saatkamp [1], Nicholas Jäck [1], Janita Müller [1], Alexander K. Strzelczyk [1], Stephan Schmidt [1,*]

[1] Institute for Organic and Macromolecular Chemistry, Heinrich-Heine-University, Universitätsstr. 1 40225 Düsseldorf Germany; Fabian.Schroeer@hhu.de

* Stephan.Schmidt@hhu.de

Contents

Video S1. Bacterial mobility: time-lapse movies 2

Table S1. FIJI macro 2

Table S2. Microgel synthesis 3

Figure S1. Glycomonomer synthesis 3

4.1 2'-acrylamidoethyl-2,3,4,6-tetra-O-acetyl-ß-D-galactopyranose (AcGalEAm) 3

4.2 1.2 2'-acrylamidoethyl-2,3,4,6-tetra-O-acetyl-α-D-mannopyranoside (AcManEAm) 7

Figure S2. Calibration curve - phenol sulfuric acid method 9

Table S3. Bacteria and buffer 9

E. Coli PKL 1162 9

LB-medium 9

PBS buffer 9

Lectin binding buffer (LBB) 10

Table S4. Instruments 10

Nuclear Magnetic Resonance spectroscopy (NMR) 10

UV-Vis spectroscopy 10

Supporting references 10

Video S1. Bacterial mobility: time-lapse movies

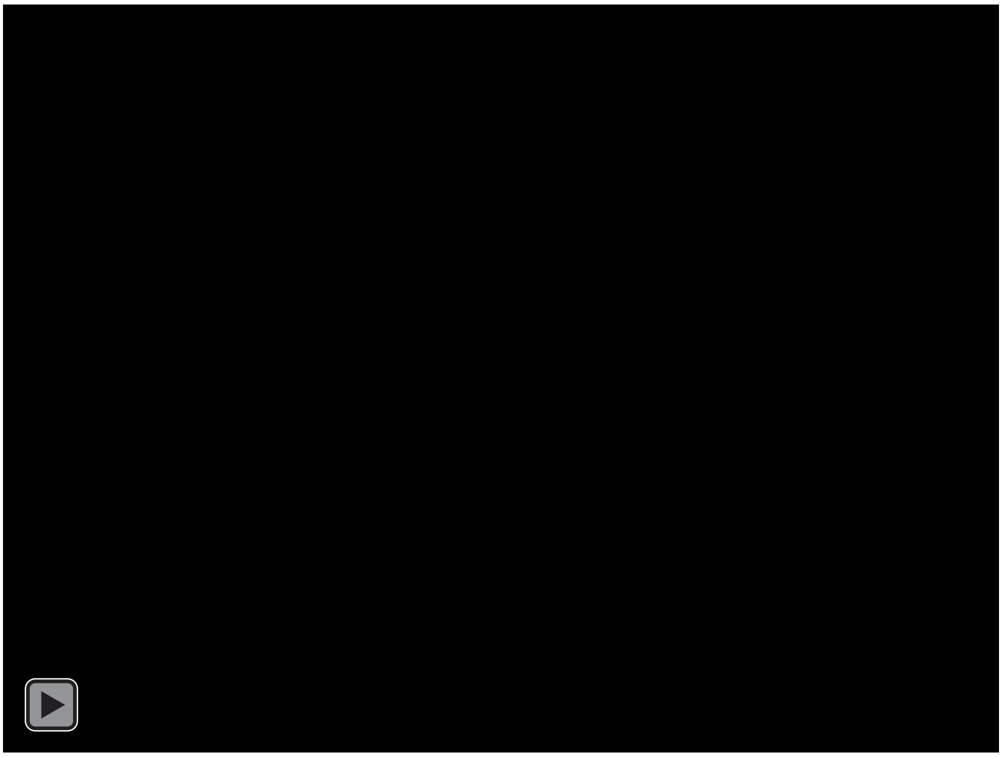

Table S1. FIJI macro

The picture stacks were analyzed with a FIJI[1] macro. In a first step all pictures were set to 8-bit. In a second step the average grey intensity of the stack was measured in the ROI. In a third step the pixel-wise gray value difference of consecutive pictures in the stack was calculated. A higher bacteria movement leads to a brighter difference picture. In a fourth step the grey intensity of the difference picture is measured at the same ROI. The used macro produces two numbers, the average grey intensity (aG) and the average difference (aD). The average difference is divided by the average grey intensity to get a value that represents bacteria movement regardless of the number of bacteria at the region of interest.

The following lines show the used FIJI macro:

```
run("8-bit");

run("Z Project...", "projection=[Average Intensity]");

roiManager("Measure");

close();

run("Stack Difference", "gap=1");

run("Z Project...", "projection=[Average Intensity]");

roiManager("Measure");

close();
```

Table S2. Microgel synthesis

Table 1 Used chemicals for the synthesis of the different microgel samples

microgel sample	MEO_2MA	OEGMA	Crosslinker	Sugar-monomer	SDS	APS
Man135-EGDMA	1.500 ml (8.1 mmol)	0.417 ml (0.90 mmol)	0.0172 ml (0.093 mmol)	900 mg (3.2 mmol)	10 mg (0,032 mmol)	57 mg (0,25 mmol)
Man40-EGDMA	1.500 ml (8.1 mmol)	0.417 ml (0.90 mmol)	0.0172 ml (0.093 mmol)	200 mg (0.72 mmol)	10 mg (0,032 mmol)	57 mg (0,25 mmol)
Man57-PEGDMA550	1.500 ml (8.1 mmol)	0.417 ml (0.90 mmol)	0.0450 ml (0.091 mmol)	200 mg (0.72 mmol)	10 mg (0,032 mmol)	57 mg (0,25 mmol)
Man60-PEGDMA750	1.500 ml (8.1 mmol)	0.417 ml (0.90 mmol)	67.60 mg (0.090 mmol)	200 mg (0.72 mmol)	10 mg (0,032 mmol)	57 mg (0,25 mmol)
Gal26-EGDMA	1.500 ml (8.1 mmol)	0.417 ml (0.90 mmol)	0.0172 ml (0.093 mmol)	150 mg (0.54 mmol)	10 mg (0,032 mmol)	57 mg (0,25 mmol)
PEG-EGDMA	1.500 ml (8.1 mmol)	0.417 ml (0.90 mmol)	0.0172 ml (0.093 mmol)	-	10 mg (0,032 mmol)	57 mg (0,25 mmol)

Figure S1. Glycomonomer synthesis

4.1 2'-acrylamidoethyl-2,3,4,6-tetra-O-acetyl-ß-D-galactopyranose (AcGalEAm)

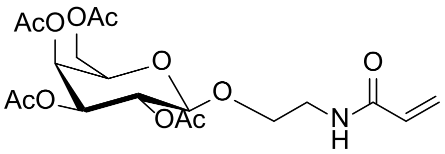

Scheme S1a AcGalEAm

The synthesis of 2'-acrylamidoethyl-2,3,4,6-tetra-O-acetyl-ß-D-galactopyranose (AcGalEAm) is based on the work of Gibson et al[2]. 6,2 g (52,2 mmol) N-(2-Hydroxyethyl)-acrylamid and 20,6 g (51,7 mmol) 1,2,3,4,6-Penta-O-acetyl-β-D-Galactose are dissolved in 600 ml dichloromethane in a 1000 ml three-neck-flask. The reaction solution is cooled down to 0 °C and purged with nitrogen for 15 min. After a slow addition of 35 ml (278,7 mmol) boron trifluoride ethyl etherate, the solution is stirred for 48 h at room temperature. The organic layer is separated and washed with ice water, two times with saturated sodium hydrogen carbonate solution, brine and with ultrapure water. The washed organic layer is dried over magnesium sulfate. Dichloromethane is removed by distillation under vacuum and reduced pressure. The synthesized AcGalEAm is purified by flash chromatography (Gradient: ethyl acetate/n-hexane 1:1 to pure n-hexane within 20 min). The yield of the remaining product is 14 % (3.63 g, 8.2 mmol).

^{1}H NMR (600 MHz, $CDCl_3$) δ 6.32 – 6.21 (m, 1H, -C=CH2), 6.07 – 6.00 (m, 1H, -C=CH2), 5.66 – 5.56 (m, 1H), 5.39 – 5.32 (m, 1H, -C=CH2), 5.32 – 5.20 (m, 1H, H4), 5.14 – 5.06 (m, 1H, H2), 4.98 – 4.90 (m, 1H, H3), 4.47 (dd, *J* = 70.5, 7.9 Hz, 1H, H1), 4.12 – 4.04 (m, 2H, H6), 4.04 – 4.00 (m, 1H, H5), 3.87 – 3.83 (m, 1H, -OCH_2**CH_2**NH), 3.74 – 3.63 (m, 1H, -OC**H_2**CH_2NH), 3.58 – 3.52 (m, 1H, -OCH_2C**H_2**NH), 3.43 (m, 1H, -OCH_2C**H_2**NH)2.11 – 2.08 (m, 3H, -$COCH_3$), 2.00 – 1.98 (m, -$COCH_3$), 1.98 – 1.97 (m, 3H, -$COCH_3$), 1.94 – 1.92 (m, 3H, -$COCH_3$).

^{13}C NMR (600 MHz, $CDCl_3$) δ 170.41 (1C, -**C**OCH_3), 170.18 (1C, -**C**OCH_3), 170.08 (1C, -**C**OCH_3), 169.81 (1C, -**C**OCH_3), 165.47 (1C, -CONH), 130.69 (1C, COCHCH$_2$), 126.72 (1C, COCH**C**H_2), 101.49 (1C, C1), 70.89 (1C, C2), 70.69 (1C, C3), 69.15 (1C, C4), 68.97 (1C, C5), 66.98 (1C, -O**C**H_2CH_2), 61.40 (1C, C6),

39.18 (1C, NH**C**H_2CH_2), 20.86 (1C, -CO**C**H_3), 20.69 (1C, -CO**C**H_3), 20.67 (1C, -CO**C**H_3), 20.58 (1C, -CO**C**H_3).

MS for $C_{19}H_{27}NO_{11}$ (ESI) *m/z* [M+ H^+]$^+$ calc. 446.2; found 446.2, [M+ Na^+]$^+$ calc.: 468.1; found 468.2.

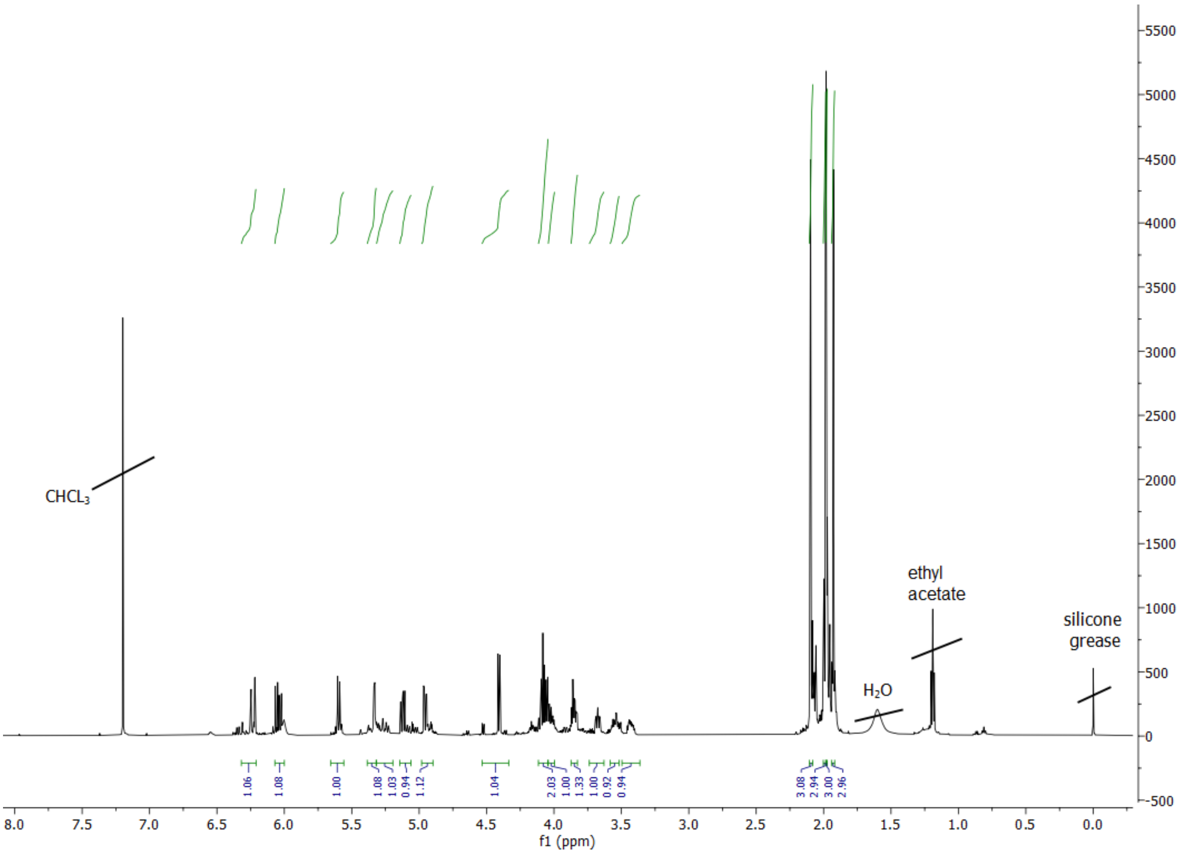

Figure 1 ^1H-NMR (600 MHz, $CDCl_3$) AcGalEAm.

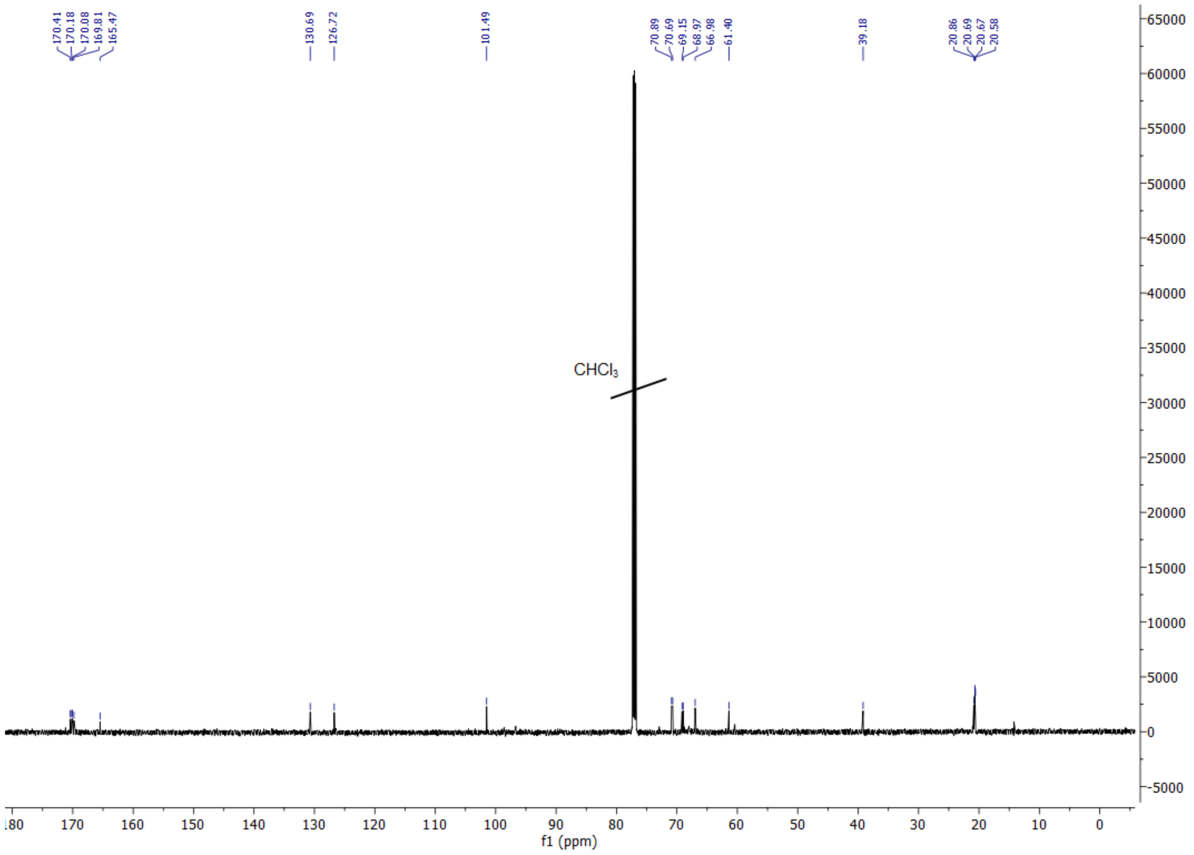

Figure 2 ^{13}C-NMR (600 MHz, $CDCl_3$) AcGalEAm.

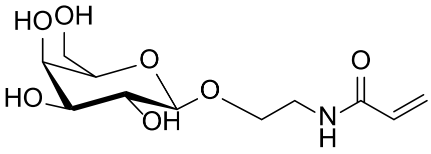

Scheme S1b GalEAm

The sugar monomer must be deprotected for the polymerization. For the deprotection, 0.85 g of the protected monomer AcGalEAm is shaken in about 15 ml of a 0.3 M sodium methanolate solution for 4 h. The formed precipitate is washed with methanol and purified by distillation under vacuum and reduced pressure. The yield of the remaining product is 28 % (0.15 g, 0.54 mmol).

^{1}H NMR (600 MHz, DMSO-d6) δ 8.10 (1H, NH), 6.24 (1H, -C=CH_2), 6.08 (1H, -C=CH_2), 5.58 (1H, -C=CH_2), 4.88 (1H, H1), 4.71 (1H, OH), 4.60 (1H, OH), 4.52 (1H, OH), 4.35 (1H, OH), 4.10 (1H, H5), 3.76 (1H, H2), 3.72 – 3.60 (m, 2H, -$OCH_2C\mathbf{H}_2NH$), 3.58 – 3.47 (m, 4H, H2, H4, H6, H6‘), 3.18 – 3.16 (m , 2H, -$OCH_2C\mathbf{H}_2NH$).

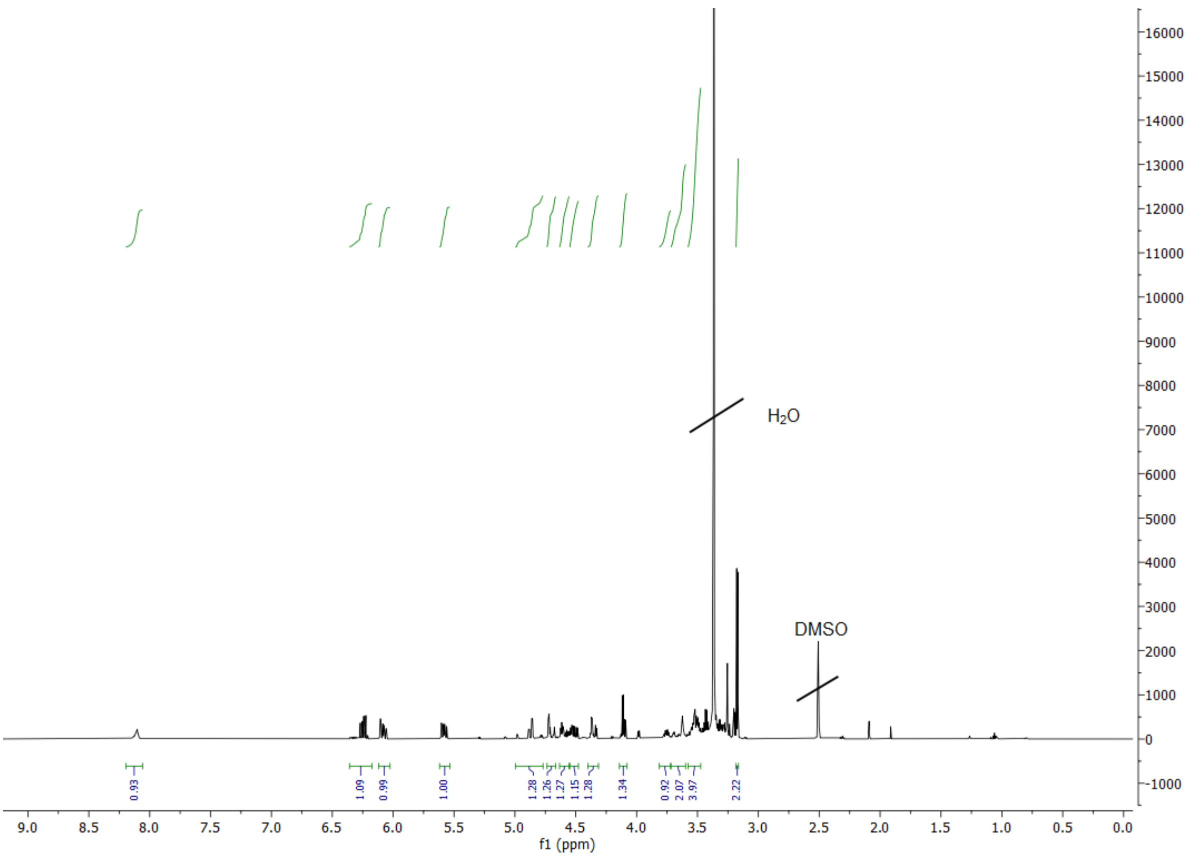

Figure 3 ^{1}H-NMR (600 MHz, DMSO-d6) GalEAm.

4.2 1.2 2'-acrylamidoethyl-2,3,4,6-tetra-O-acetyl-α-D-mannopyranoside (AcManEAm)

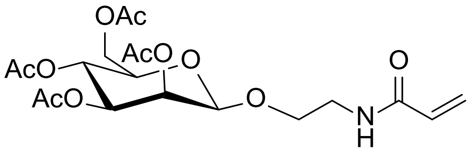

Scheme S2a AcManEAm

The synthesis of 2'-acrylamidoethyl-2,3,4,6-tetra-O-acetyl-α-D-mannopyranoside (AcManEAm) is very similar to the synthesis of AcGalEAM. 10,0 g (78,2 mmol) N-(2-Hydroxyethyl)-acrylamid and 33,4 g (85,5 mmol) 1,2,3,4,6-Penta-O-acetyl-α-D-mannopyranoside are dissolved in 700 ml of dichloromethane, cooled down to 0 °C and flushed with nitrogen for 15 min inside a 1000 ml three-neck-flask. After the slow addition of 42 ml (331,4 mmol) boron trifluoride ethyl etherate, the reaction solution is stirred at room temperature for 48 h. The organic layer is washed with with icewater, 3 times with saturated sodium hydrogen carbonate solution and with ultrapure water. After drying the orangic layer over magnesium sulfate, dichloromethane is removed by distillation under vacuum and reduced pressure. The synthesized AcManEAm is purified by column chromatography (Gradient: ethyl acetate/n-hexane 1:1 to pure n-hexane). The yield of the remaining product is 52 % (18,0 g, 40 mmol).

^{1}H NMR (600 MHz, DMSO-d_6) δ 8.31 (t, J = 5.7 Hz, 1H, NH), 6.25 (dd, J = 17.1, 10.2 Hz, 1H, -CH=CH_2), 6.09 (dd, J = 17.1, 2.2 Hz, 1H, -CH=CH_2), 5.60 (dd, J = 10.2, 2.2 Hz, 1H, -CH=CH_2), 5.20 – 5.15 (m, 1H, H2), 5.14 (dd, J = 3.6, 1.6 Hz, 1H, H3), 5.11 – 5.04 (m, 1H, H4), 4.89 (d, J = 1.7 Hz, 1H, H1), 4.12 (dd, J = 12.2, 5.3 Hz, 1H, H6), 4.04 – 4.01 (m, 1H, H6‘), 4.01 – 3.97 (m, 1H, H5), 3.71 – 3.64 (m, 1H, -$OCH_2C\mathbf{H}_2NH$), 3.57 – 3.51 (m, 1H, -$OC\mathbf{H}_2CH_2NH$), 3.41 – 3.34 (m, 2H, -$OCH_2C\mathbf{H}_2NH$), 2.11 (s, 3H, -$COCH_3$), 2.03 (s, 3H, -$COCH_3$), 2.02 (s, 3H, -$COCH_3$), 1.94 (s, 3H, -$COCH_3$).

^{13}C NMR (600 MHz, $CDCl_3$) δ 170.67 (1C, -**C**OCH_3), 170.12 (1C, -**C**OCH_3), 170.12 (1C, -**C**OCH_3), 169.70 (1C, -**C**OCH_3), 165.62 (1C, -CONH), 130.54 (1C, CO**C**HCH_2)), 126.98 (1C, COCH**C**H_2), 97.77 (1C, C1), 69.37 (1C, C2), 68.99 (1C, C3), 68.79 (1C, C4), 67.59 (1C, C5), 66.16 (1C, -O**C**H_2CH_2), 62.52 (1C, C6), 39.14 (1C, NH**C**H_2CH_2), 20.88 (1C, -CO**C**H_3), 20.73 (1C, -CO**C**H_3), 20.71 (1C, -CO**C**H_3), 20.71 (1C, -CO**C**H_3).

MS for $C_{19}H_{27}NO_{11}$ (ESI) *m/z* [M+ H^+]$^+$ calc. 446.2; found 446.1, [M+ Na^+]$^+$ calc.: 468.1; found 468.2.

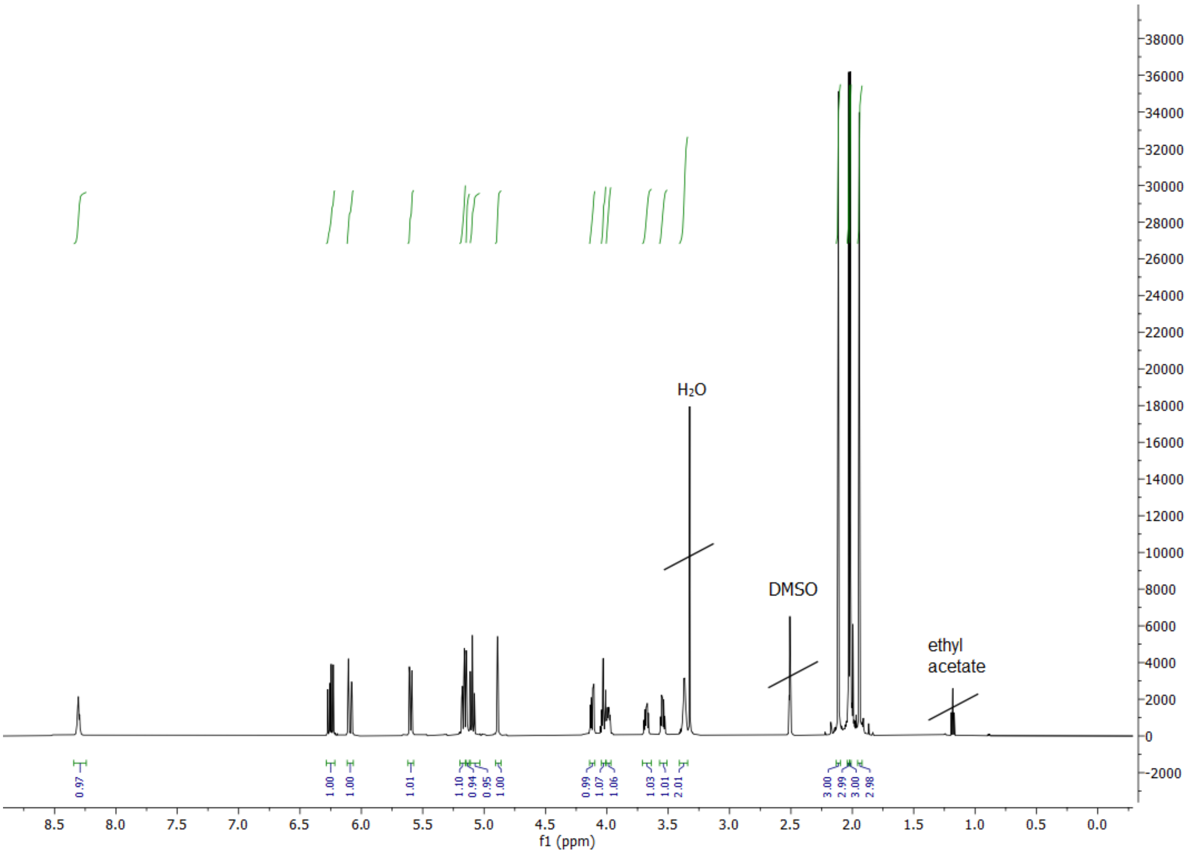

Figure 4 ^{1}H-NMR (600 MHz, DMSO-d_6) AcManEAm.

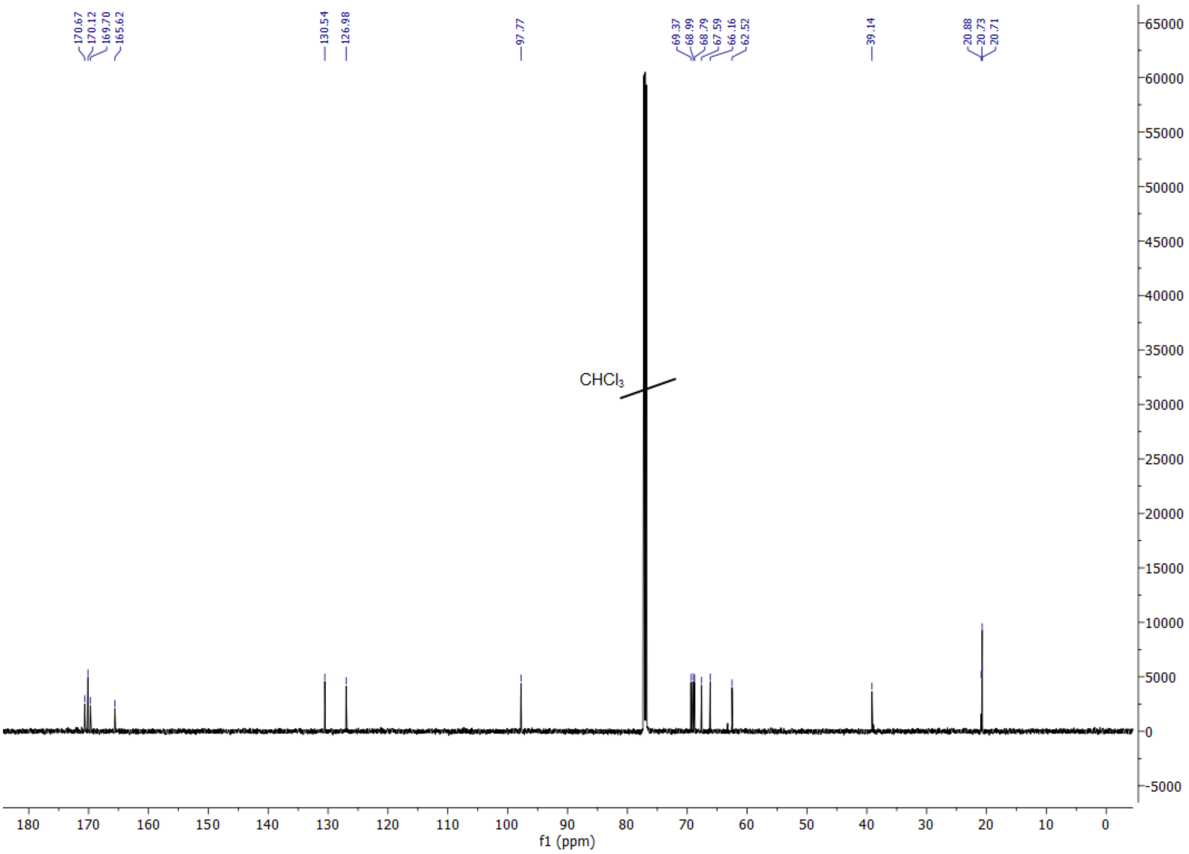

Figure 5 ^{13}C-NMR (600 MHz, $CDCl_3$) AcManEAm.

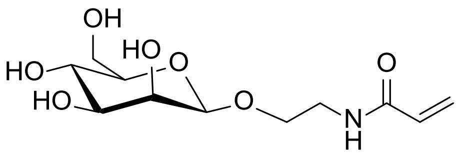

Scheme S2b ManEAm

The deprotection process of AcManEAm is the same process described for the deprotection of AcGalEAm.

Figure S2. Calibration curve - phenol sulfuric acid method

For the calibration curve of the phenol sulfuric acid method the amount of Methyl α-D-mannopyranoside was varied between 0 and 500 µM. The absorption was determined at 490 nm.

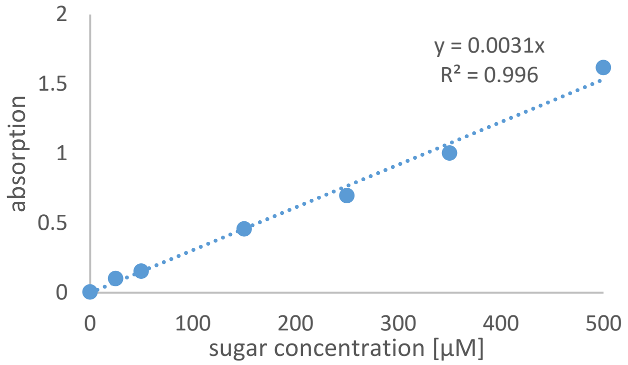

Figure 6 Calibration curve of the phenol sulfuric acid method

Table S3. Bacteria and buffer

E. Coli PKL 1162

Overnight, E. coli PKL 1162 are grown in LB medium (PKL 1162) in a sterilized test tube, at 40 °C. The tubes are shaken with a speed of 120 rpm.

LB-medium

LB-medium contains 12.5 g of LB Broth (Miller) which is dissolved in 500 mL ultrapure water. This so-called powder microbial growth medium contains tryptone (5.0 g), sodium chloride (5.0 g) and yeast extract (2.5 g). This solution is sterilized for about 30 min at 121 °C and cooled to room temperature. Finally, 50.0 mg of ampicillin and 25.0 mg of chloramphenicol are added.

PBS buffer

One tablet of phosphate buffered saline is dissolved in 0.2 L of ultrapure water. The pH is adjusted to 7.4 with 1 M NaOH. The final concentration of the buffer is 137 mM sodium chloride, 2 mM potassium chloride and 10 mM phosphate buffer.

Lectin binding buffer (LBB)

The Lectin binding buffer (LBB), which is used for all Concanavalin A measurements, contains 10 mM HEPES ((4-(2-hydroxyethyl)-1-piperazineethanesulfonic acid) as a buffering agent. After adjusting the pH to 7.4 with 1 M NaOH, 1 mM calcium chloride and 1 mM manganese chloride is dissolved in the solution.

Table S4 Instruments

Nuclear Magnetic Resonance spectroscopy (NMR)

A Bruker AVANCE III -600 (Bremen, Germany) is used to perform ^{1}H-NMR and ^{13}C-NMR (600 MHz) measurements. As a solvent $CDCl_3$ and DMSO-d_6 is used. Referring to internal standard, for the ^{1}H-NMR and ^{13}C-NMR the signals of the remaining protons of the deuterated solvent is used (CDCl3: 1H 7.20, 13C 77.24, DMSO-d6: 1H 2.50). The chemical shifts, which are reported in delta (δ), are expressed in parts per million (ppm). The conventional abbreviations s (singlet), d (doublet), t (triplet), q (quartet), dd (doublet of doublets), m (multiplet), are used.

UV-Vis spectroscopy

A Specord® 210 Plus UV-Vis photometer from Analytik Jena AG (Jena, Germany) is used to perform all UV-Vis measurements. The Win ASPECT PLUS software is used for instrument operation. All measurements are performed at 20 °C. The phenol sulfuric acid-method and E. Coli concentration measurements are performed in Polystyrene cuvettes (d = cm, V = 2.5 ml) from Sarstedt (Nümbrecht, Germany). The spectral scan range for the phenol sulfuric acid-method is 300 to 550 nm, for E. Coli concentration measurements 550 to 650 nm. All measurements are performed at 20 °C. Con A concentration measurements are performed in a QX quartz cuvette (d = 1 cm, V = 3.5 mL) from Hellma Anayltics (Mühlheim, Germany). The spectral scan range is 250 to 300 nm.

Supporting references

1. Schindelin, J.; Arganda-Carreras, I.; Frise, E.; Kaynig, V.; Longair, M.; Pietzsch, T.; Preibisch, S.; Rueden, C.; Saalfeld, S.; Schmid, B.; Tinevez, J.-Y.; White, D. J.; Hartenstein, V.; Eliceiri, K.; Tomancak, P.; Cardona, A., Fiji: an open-source platform for biological-image analysis. *Nature Methods* **2012,** *9* (7), 676-682.
2. Wilkins, L. E.; Phillips, D. J.; Deller, R. C.; Davies, G.-L.; Gibson, M. I., Synthesis and characterisation of glucose-functional glycopolymers and gold nanoparticles: study of their potential interactions with ovine red blood cells. *Carbohydrate Research* **2015,** *405*, 47-54.
